# EMBRACING COMMUNITY: ADVANCE CARE PLANNING FOR AFRICAN AMERICAN OLDER ADULTS LIVING WITH DEMENTIA

**DOI:** 10.1093/geroni/igac059.1481

**Published:** 2022-12-20

**Authors:** Karen Moss, Kathy Wright, Emika Miller, Kimberly Lawson, Karen Rose, Todd Monroe, Celia Wills

**Affiliations:** The Ohio State University, Columbus, Ohio, United States; The Ohio State University College of Nursing, Columbus, Ohio, United States; Ohio State University, Columbus, Ohio, United States; African American Alzheimer's and Wellness Association, Westerville, Ohio, United States; The Ohio State University, Columbus, Ohio, United States; Ohio State University College of Nursing, Columbus, Ohio, United States; Ohio State University College of Nursing, Columbus, Ohio, United States

## Abstract

Lower socioeconomic status African American older adults living with dementia and their family caregivers face unique advance care planning challenges, including achieving appropriate preference-consistent healthcare near the end of life. The purpose of this project within a larger multi-stakeholder study is to assess community stakeholder perspectives on the needs of African American older adults living with dementia and their family caregivers. Diverse community-based organization leaders (n=9) were interviewed and identified the following thematic needs: Reframing of Advance Care Planning, Clarification of Misinformation, and Expansion of Available Resources. Community leaders expressed strong desires to assist families with advance care planning. The use of creative, culturally-tailored, non-traditional approaches to this process offers innovative ways to promote advance care planning through community engagement. This will change the narrative on advance care planning from a cultural perspective while embracing diversity, enriching discovery, and reimaging aging in the community

